# Biogenic synthesis and characterization of gold nanoparticles by *Escherichia coli* K12 and its heterogeneous catalysis in degradation of 4-nitrophenol

**DOI:** 10.1186/1556-276X-8-70

**Published:** 2013-02-12

**Authors:** Sarvesh Kumar Srivastava, Ryosuke Yamada, Chiaki Ogino, Akihiko Kondo

**Affiliations:** 1 Department of Chemical Science and Engineering, Graduate School of Engineering, Kobe University, 1-1 Rokkodai-cho, 657-8501, Nada, Kobe, Japan; 2 Organization of Advanced Science and Technology, Kobe University, 1-1 Rokkodai-cho, 657-8501, Nada, Kobe, Japan

**Keywords:** Gold nanoparticles, Extracellular biosynthesis, Green catalysis, *Escherichia coli*, Nitrophenol degradation, Water treatment

## Abstract

Room-temperature extracellular biosynthesis of gold nanoparticles (Au NPs) was achieved using *Escherichia coli* K12 cells without the addition of growth media, pH adjustments or inclusion of electron donors/stabilizing agents. The resulting nanoparticles were analysed by ultraviolet–visible (UV–vis) spectrophotometry, atomic force microscopy, transmission electron microscopy and X-ray diffraction. Highly dispersed gold nanoplates were achieved in the order of around 50 nm. Further, the underlying mechanism was found to be controlled by certain extracellular membrane-bound proteins, which was confirmed by Fourier transformation-infrared spectroscopy and sodium dodecyl sulfate polyacrylamide gel electrophoresis. We observed that certain membrane-bound peptides are responsible for reduction and subsequent stabilization of Au NPs (confirmed by zeta potential analysis). Upon de-activation of these proteins, no nanoparticle formation was observed. Also, we prepared a novel biocatalyst with Au NPs attached to the membrane-bound fraction of *E. coli* K12 cells serving as an efficient heterogeneous catalyst in complete reduction of 4-nitrophenol in the presence of NaBH_4_ which was studied with UV–vis spectroscopy. This is the first report on bacterial membrane-Au NP nanobiocomposite serving as an efficient heterogeneous catalyst in complete reduction of nitroaromatic pollutant in water.

## Background

Gold nanoparticle (Au NP), being the most stable mono-metallic nanoparticle, promises to be a key material and building block for newer technologies in the twenty-first century. Gold in its bulk state is regarded as a noble metal and is very unreactive because of its completely filled d-band [1]. However, at nanoscale, it is proving to be an important material for catalysis owing to its shape, size and crystal structure arrangement [2]. Due to this new set of properties, it has found wide-scale application in optics, electronics, catalysis, fabrication and biomedical utilities [3]. Generally speaking, physical methods of producing gold nanoparticles involve heating of gold at reduced pressure to generate gold vapour, while chemical synthesis requires a reducing agent (generally citrate) followed by addition of a stabilizing agent [4-7]. However, these chemical methods deliver at the cost of expensive reducing and capping agents and toxic solvents along with tedious process control. To overcome these issues, several biogenic synthesis processes have been reported owing to the constant need for cost-effective eco-friendly synthesis of Au NPs. Microbial systems have found an important role in nanoparticle production due to their natural mechanism for detoxification of metallic ions through reduction which can be achieved extracellularly or intracellularly through bioaccumulation, precipitation, biomineralization and biosorption. Ogi et al. [8] showed gold nanoparticle formation in the presence of H_2_ gas pumped with *Shewanella algae* cell extract. Similarly, gold nanoparticles of different shapes and sizes were produced using bacterial and fungal strains [9-12]. However, apart from the stated advantages, biological synthesis suffers from poor mono-dispersity, random aggregation, non-uniform shapes, problems in scale-up, etc. [13]. Though, in recent times, many organisms have been reported to produce nanoparticles, scientific understanding on the mechanism and the machinery related to its production is still in its infancy. Therefore, there is a need to improve upon this green synthesis process with an aim to understand the underlying mechanism and design a working prototype for biomimetic production of Au NPs.

These nanoparticles, upon being adhered to a matrix, may serve as a better catalyst than bulk metal due to greater accessibility to surface atoms and low coordination number especially in the case of water treatment. Among several water pollutants, nitroaromatic compounds are considered as the most toxic and refractory pollutants, of which the permissible range is as low as 1 to 20 ppb. However, these are common in production of dyes, explosives and pesticides among many others; thus, their industrial production is considered as an environmental hazard [14]. Upon being released into the environment, these nitrophenols pose significant public health issues by exhibiting carcinogenic and mutagenic potential in humans [15].

Normally, it takes a long time for degradation of nitrophenols in water which poses considerable risk if it seeps into aquifers along with the groundwater. These nitrophenols tend to get accumulated in deep soil and stays indefinitely. Although several water treatment methods are available like chemical precipitation, ion exchange adsorption, filtration and membrane systems, they are slow and non-destructive. Therefore, there is a need to remove these highly toxic compounds with efficient catalytic systems. Generally, nanoparticles are immobilized onto supporting materials like silica, zeolites, resins, alumina, microgels, latex, etc. which are inert to the reactants and provide a rigid framework to the nanoparticles. The gold-supported catalysts can then be used to carry out partial or complete oxidation of hydrocarbons, carbon monoxide, nitric oxide, etc. [16]. In a recent study, Deplanche et al. [17] showed coating of palladium followed by gold over *Escherichia coli* surface in the presence of H_2_ to produce biomass-supported Au-Pd core-shell-type structures and subsequent oxidation of benzyl alcohol. Likewise, we believe that bacterial biomass is essentially carbonaceous matter which can be used to serve as a matrix for preparing a heterogeneous catalyst with the incorporation of nanoparticles. With this aim, we utilized *E. coli* K12 strain to check its potential for producing Au^0^ from AuCl_4_ ^−^. This strain has been known for its reduction activity as shown with bioremediation studies [18,19]. Here, we report bioreduction of gold cations at room temperature to yield well-dispersed nanoparticles (Au^0^). The resulting nanoparticles were characterized by ultraviolet–visible (UV–vis) spectroscopy, atomic force microscopy (AFM), selected-area electron diffraction (SAED), transmission electron microscopy (TEM) and X-ray diffraction (XRD). Additionally, the extracellular reduction mechanism was examined by Fourier transformation-infrared spectroscopy (FT-IR), zeta potential (Z-pot) and sodium dodecyl sulfate polyacrylamide gel electrophoresis (SDS-PAGE). We observed that certain membrane-embedded proteins in the extracellular membrane fraction of the cell are responsible for reducing gold cation to stable Au^0^ state. Further, these membrane-bound gold nanoparticles were utilized to produce a heterogeneous catalyst in degradation of 4-nitrophenol (4-NP). This biosynthesis study provides an excellent platform for the production of gold nanoparticles by bacterial membrane-bound proteins. The resulting membrane-bound nanoparticles can be prepared into an eco-friendly cost-effective bionanocomposite to serve as an efficient catalyst in complete degradation of 4-nitrophenol.

## Methods

### Bacterial strain and growth conditions

*E. coli* K12 cells were procured from our existing strain collection and were cultured in nutrient broth (10 g L^−1^ peptone, 10 g L^−1^ meat extract, 0.5 g L^−1^ NaCl) at 27°C and 120 rpm for 24 h in screw-capped flasks. After a day of incubation, the culture was centrifuged at 10,000×*g* for 10 min, and the resulting bacterial pellet was separated and retained. The bacterial pellet was thoroughly washed three times in sodium saline followed by washing three times in Milli-Q water (Millipore, Tokyo, Japan) to remove any unwanted material sticking to the cells. These cells were weighed, and 0.5 g wet weight of pellet was prepared to be used later. The washed cells suspended in 10 mL of distilled water gave a solution with a cell concentration of 5.2 × 10^11^ cells mL^−1^.

To determine whether or not intact cells were required for Au NP formation, *E. coli* K12 cells were cultured and harvested as in the previously described method. The cells were then disrupted by autoclaving (120°C at 15 psi for 30 min). This caused complete lysis of the bacterial cells which were later centrifuged at 15,000×*g* for 60 min to separate the membrane fraction (pellet) from the soluble (supernatant) fraction. Membrane-bound fraction (MBF) pellet was pooled together and washed thrice with Milli-Q water and re-centrifuged again at 15,000×*g* for 30 min. Finally, 2 g of MBF pellet (wet wt.) was retained to be incorporated with 10 mL of 0.01 M HAuCl_4_ solution (Nacalai Tesque, Kyoto, Japan). Although pH was measured at this stage (pH 2.8), no adjustment was made. Control reactions included 0.01 M HAuCl_4_ solution prepared with soluble (supernatant) fraction and uninoculated HAuCl_4_ solution prepared with Milli-Q water.

### Characterization of biogenic gold nanoparticles

UV-visible spectra were obtained using a Hitachi U2000A UV–vis spectrophotometer (Tokyo, Japan). The formation of Au NPs was monitored by UV–vis spectra of the reaction mixture from 210 to 800 nm.

Primary study of nanoparticle shape and size was carried out using an SPI-3800N atomic force microscope with SPA 400 soundproof housing sample holder connected to an imaging system (Seiko Instruments, Chiba, Japan). Five microlitres was taken from the reaction mixture and placed on the glass grid and dried at room temperature. The images were obtained using SPIWin (3800N) ver. 3.02J (Wyandotte, MI, USA).

Morphology and grain size of these nanoparticles were analysed using a Hitachi H-7100 transmission electron microscope. Two microlitres was taken from the two reaction mixtures and placed on carbon-coated copper grids and dried at room temperature. The transmission electron micrographs and the SAED patterns were recorded at an acceleration voltage of 100 kV. The images were analysed using the ImageJ 1.43M software.

FT-IR analysis was done using Jasco FT/IR-680 plus (Easton, MD, USA) coupled to a high-performance computer. The samples (100 μL) were placed over the ATR analyser, and the resulting spectra were analysed using Spectra Manager ver. 1.06.02. Zeta potential measurements were performed using the Malvern Zetasizer Nano ZS model ZEN3600 (Malvern, UK) equipped with a standard 633-nm laser.

Confirmatory study of resulting Au NPs was done by XRD using a Rigaku RINT-TTR diffractometer (Tokyo, Japan) equipped with a parallel incident beam (Göbel mirror) and a vertical θ-θ goniometer. Samples were placed directly on the sample holder. The X-ray diffractometer was operated at 50 kV and 300 mA to generate CuKα radiation. The scan rate was set to 5° mil^−1^. Identification of the metallic gold was obtained from the JCPDS database.

### Preparation of biomass-supported Au nanocatalyst in 4-nitrophenol degradation

The reduction of 4-NP by NaBH_4_ was studied as a model reaction to probe catalytic efficiency of a biomass-supported Au catalyst for heterogeneous systems. Under experimental conditions, reduction does not proceed at all simply with the addition of NaBH_4_ or biomass alone. However, in the presence of a biomass-supported Au catalyst, it proceeds to completion with formation of 4-aminophenol (4-AP). To study the reaction in a quartz cuvette, 2.77 mL of water was mixed with 30 μL (10^−2^ M) of 4-NP solution and 200 μL of freshly prepared NaBH_4_ (10^−1^ M) was added. The Au NP reaction mixture along with the MBF was dried for 24 h at 90°C, and 5 mg of biomass-Au NP composite (size approximately 50 nm, 4.2 × 10^−6^ mol dm^−3^) was added to the above reaction mixture. A similar technique was used by Narayanan and Sakthivel [20] by coating fungal mycelia-coated Au NPs on glass beads. UV–vis spectra of the sample were recorded at every 2-min interval in the range of 200 to 600 nm. The rate constant of the reduction process was determined by measuring the change in absorbance of the initially observed peak at 400 nm, for the nitrophelate ion as the function of time.

## Results and discussion

### Characterization of biogenic Au NPs

**Figure 1:**
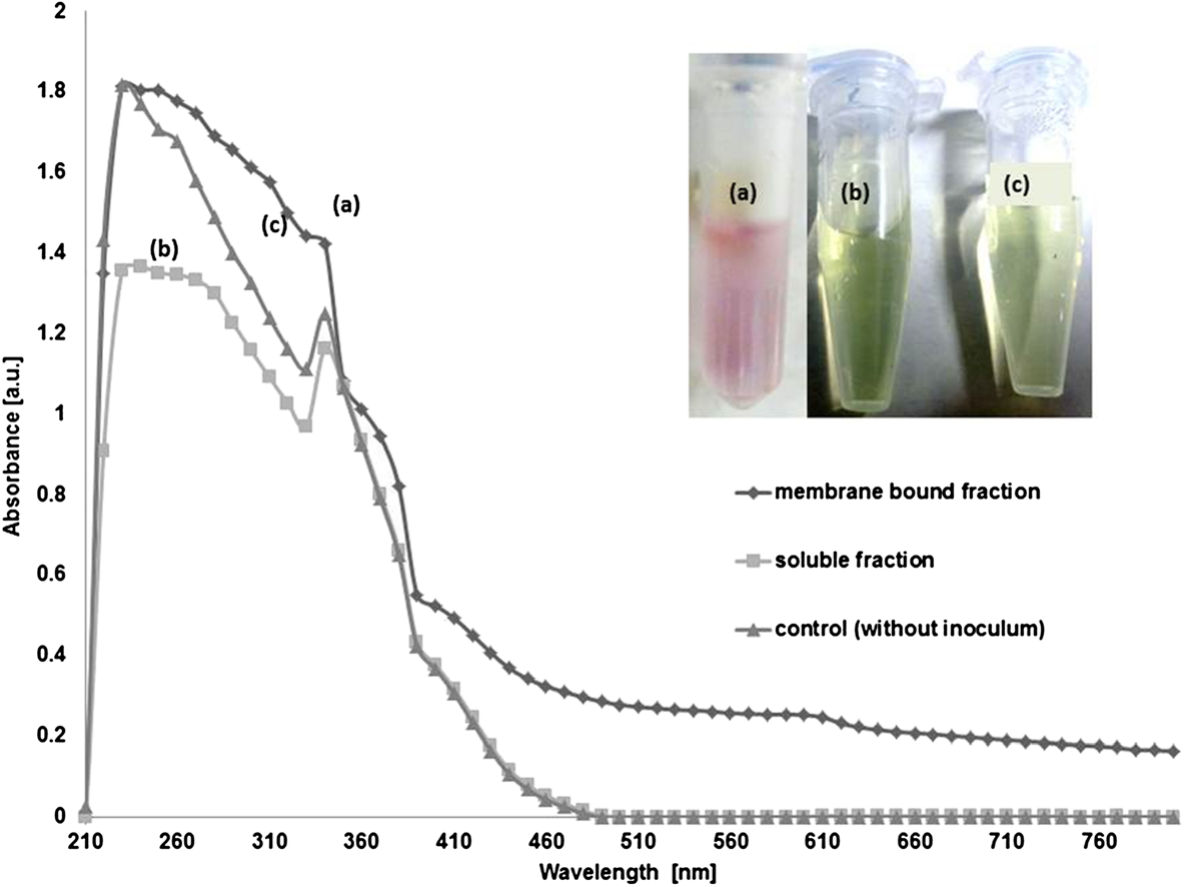
**UV–vis spectra observed after 24 h.** (**a**) SPR due to Au NP produced by MBF; (**b**) no SPR absorbance in soluble fraction; (**c**) no SPR absorbance in gold ion solution without inoculum. The inset figure corroborating the same in the above-mentioned samples, respectively.

Gold nanoparticle formation was primarily observed by UV–vis spectroscopy and AFM. After a series of experimentations, we found that MBF of *E. coli* K12 strain has certain proteins which are responsible for reducing Au cations into Au NPs. A distinct pink colour was observed due to the phenomenon of surface plasmon resonance (SPR) [21] (Figure 1a) in the reaction mixture containing MBF of the bacterial cell after 24 h. No colour formation was present in the control sample consisting of soluble fraction (Figure 1b) and gold ion solution without inoculum (Figure 1c). The same is shown in the inset of Figure 1. UV–vis spectra (Figure 1) of aqueous reaction mixtures showed no increase in absorbance after 24 h, suggesting formation of stable nanoparticles in the reaction mixture. It should be noted that the SPR peak broadening and associated decreased intensity is because of the interaction between the membrane fraction and Au NPs in the reaction mixture. [22] This can be understood by the fact that when these Au NPs are in the vicinity of bacterial cells, membrane fraction or lipopolysaccharides, they tend to adhere to these substrates, thereby reducing the peak intensity (adding scattering background) as compared to otherwise observed SPR of Au NPs alone. This also suggests that in the case of biogenic synthesis of nanoparticles, the presence and intensity of SPR should not be the sole criterion for concentration assessment.

**Figure 2:**
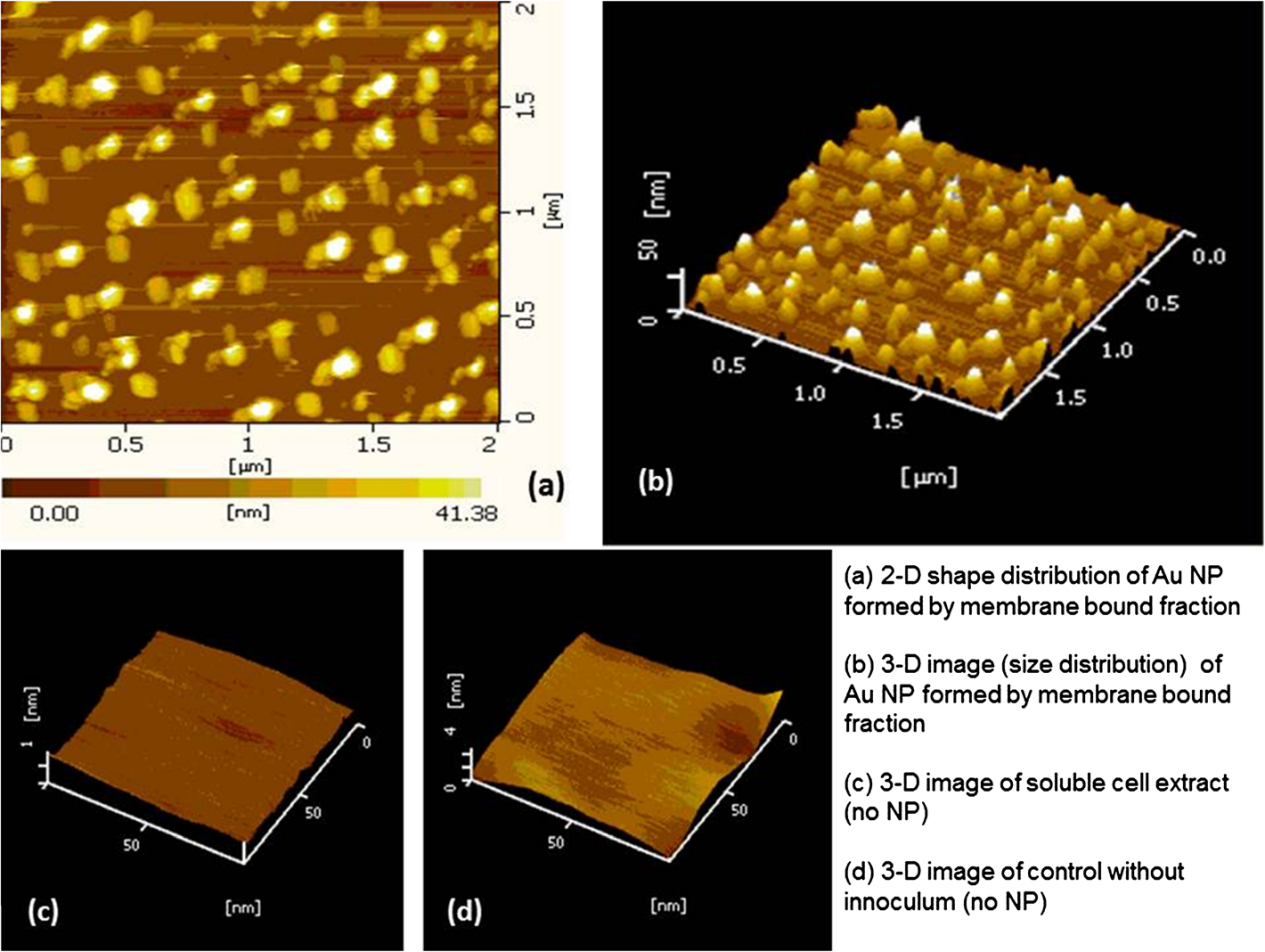
AFM imaging of biogenic Au nanospheres after 24 h by membrane-bound fraction of cells (a-d).

It is important to note that no colour change was observed in control solutions consisting of cell soluble fraction and gold cation solution (without inoculum), suggesting the absence of nanoparticle formation. This was further verified when these samples were examined by AFM as shown in Figure 2.

**Figure 3:**
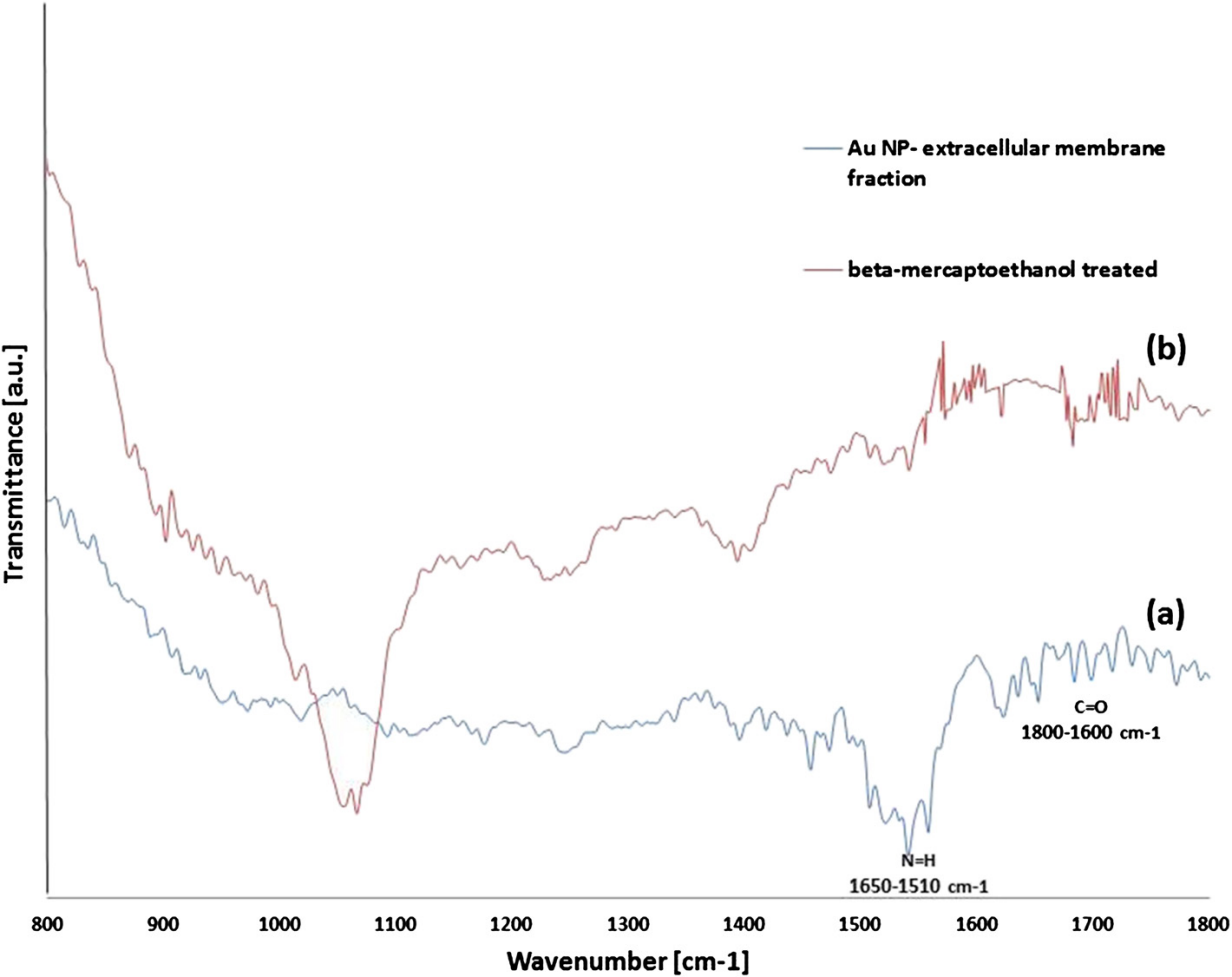
**FT-IR spectra of reaction solution.** (**a**) Membrane-bound fraction with Au NPs (indicated in blue); (**b**) membrane-bound fraction treated with β-mercaptoethanol (indicated in red).

The AFM probe detected discrete circular nanoparticles (Figure 2a,b) from the MBF reaction mixture, while no such formation was observed in the soluble fraction or gold cation solution without inoculum (Figure 2c,d). The 2D profile obtained by AFM suggested strong shape control (circular) with a size around 50 nm. This strong shape control indicated that apart from reducing proteins present in the MBF, certain organic groups must be acting as stabilizing agent. To investigate the same, the membrane-bound reaction mixture was subjected to FT-IR analysis to analyse the chemical groups responsible for nanoparticle synthesis. FT-IR spectra (Figure 3a) showed distinct absorption in the region 1,800 to 1,600 cm^−1^ responsible for amide linkages in the reaction mixture.

FT-IR spectra (Figure 3a) confirmed the presence of vibration bands centred at 1,841, 1,787, 1,756, 1,725, 1,692, 1,680, 1,661, 1,650, 1,634 and 1,603 cm^−1^. This highlights the presence of amide I (C=O) and amide II (N=H) groups present in the reaction mixture. It is likely that the amide carbonyl group (C=O) arises from peptide coupling in proteins from the extracellular membrane fraction of the bacterial cell. This supports the fact that the secondary amide C=O stretching which forms protein/Au bioconjugates may have a role in stabilization of nanoparticles [23]. Generally, in the case of biogenic synthesis, the presence of active chemical groups like amino, sulfhydryl and carboxylic groups plays a key role in reduction of metallic ions and subsequent formation of nano/microparticles. Since amino and carboxyl groups were detected by FT-IR, it strongly suggested towards the presence of certain proteins in the reaction medium responsible for Au NP biosynthesis. Further, aqueous stability of Au NPs were tested by zeta potential analysis. It should be noted that if active groups on biomass carry greater positive charge at low pH, it weakens the reducing power of biomass and allows AuCl_4_ ^−^ ions to get closer to the reaction site [24]. This decreases the reaction rate and causes strong biosorption between Au NPs and biomass resulting in particle aggregation. Since the bacterial cell wall of *E. coli* is negatively charged, it tends to thermodynamically favour the formation of nanoparticles at low pH as observed in our case. This was confirmed by zeta potential analysis of the Au NP solution with a mean Z-pot of −24.5 ± 3.1 mV, suggesting a stable gold colloid solution. To further investigate the role of proteins in nanoparticle formation, MBF was treated with 1% β-mercaptoethanol (β-met) and heated for 30 min at 95°C. This treatment caused disruption of disulfide bonds within the multimeric chains of peptide and eventually resulted in loss of activity. In the absence of reducing activity by membrane-bound proteins, no nanoparticle formation was observed with β-met-treated MBF. This was further verified by FT-IR analysis (Figure 3b) with disappearance of most bands around the 1,600 cm^−1^ region. The peak observed at 1,075 cm^−1^ corresponds to the thiocarbonyl group due to the addition of mercaptoethanol in the reaction mixture. This suggested that certain membrane-embedded proteins may be responsible for reducing Au^3+^ to Au nanoparticles (Au^0^). The membrane proteins responsible for nanoparticle synthesis were run along with β-met-treated membrane proteins in SDS-PAGE gel (data not shown) which confirmed the presence of different sizes of protein bands in the reaction mixture, of which 25 and 73 KDa seemed to be of importance. Upon treatment of MBF with β-met, there was absence of a protein band at 73 KDa, and subsequently, no nanoparticle formation was observed, suggesting its crucial role in the reduction process. Also, a shorter peptide (25 KDa) was found to be adhered to the synthesized nanoparticles, suggesting its role in stabilization of nanoparticles. This is in accordance with our recently reported study where we concluded that ionic reduction in some bacteria takes place due to certain proteins along the lipopolysaccharides/cell wall which reduces the metallic ions in its vicinity of the bacterial cell, thereby producing stable nanoparticles [25].

**Figure 4:**
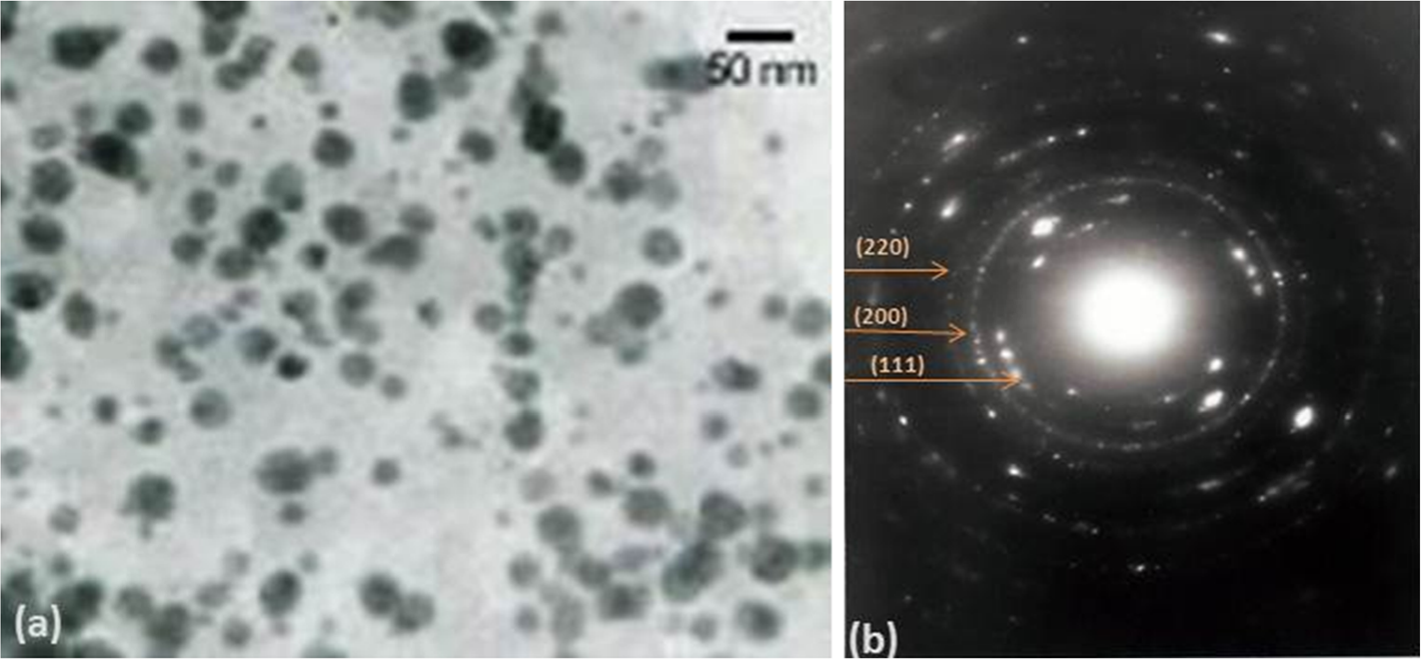
**TEM images of biogenic Au nanoparticles after 24 h.** (**a**) Discrete gold nanoparticles of size approximately 50 nm; (**b**) SAED pattern of obtained Au NPs.

Subsequently, resulting nanoparticles were analysed by TEM and XRD. TEM images (Figure 4a) confirmed the presence of discrete nanoparticles in the range of approximately 50 nm. Some small nanoparticles were also visualized suggesting inherent polydispersity as generally observed in the case of biogenic synthesis. Nanoparticle size was calculated without the encasing membrane-bound proteins. It was observed that the nanoparticles obtained were highly discrete, were circular in shape and did not show aggregation with the neighbouring particles. Also, single-crystalline structures of biogenic nanoparticles were further supported by their corresponding SAED analysis as shown in Figure 4b with characteristic {111}, {200} and {220} diffraction patterns suggesting a face-centred cube (fcc) arrangement.

**Figure 5:**
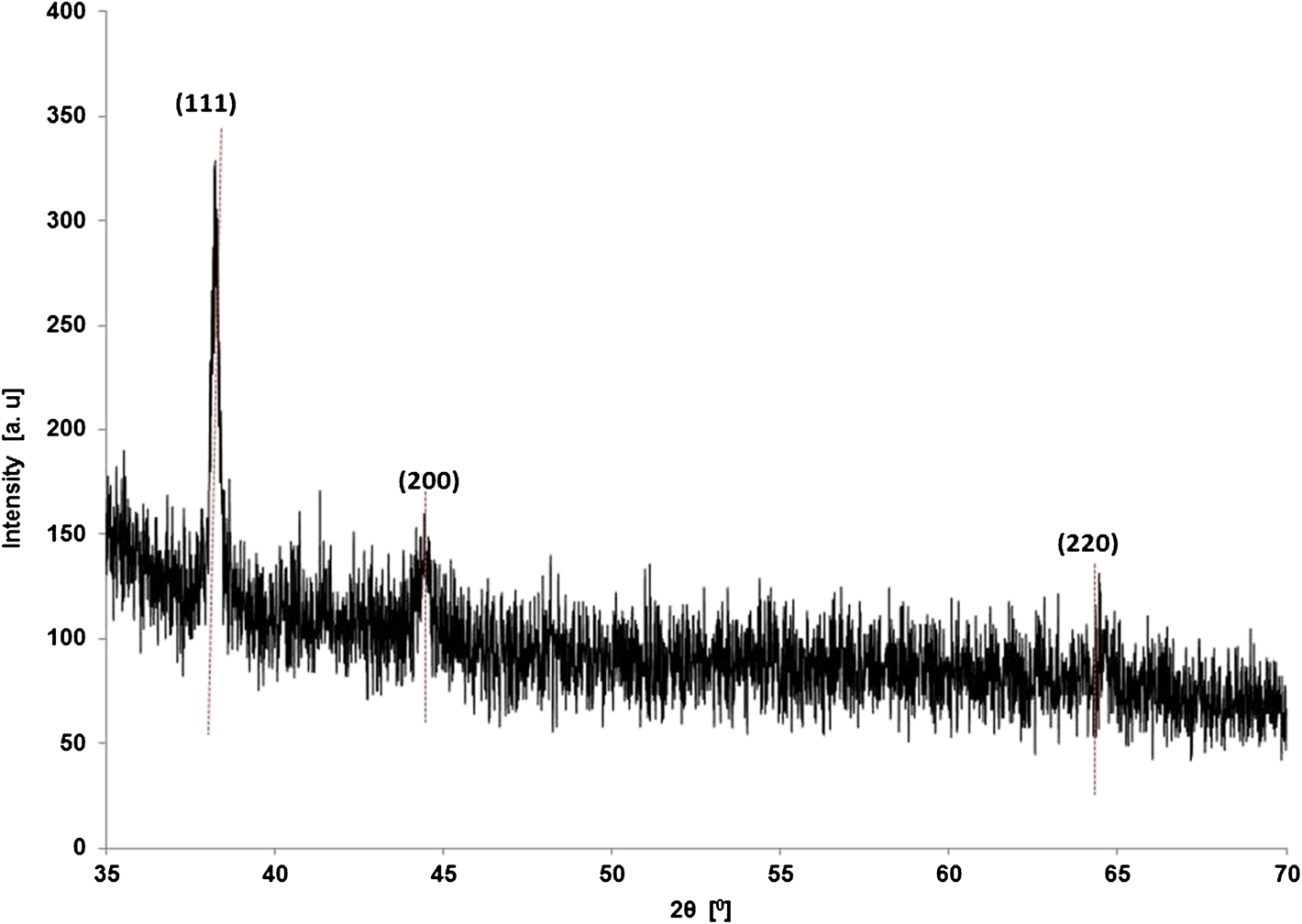
**XRD spectra of Au**^**0 **^**as obtained by membrane-bound fraction of *E. coli *K12 cells.**

Finally, confirmation of gold nanoparticles was done via XRD which confirmed the presence of synthesized gold (Figure 5). Bragg’s reflections observed in the diffraction pattern could be indexed on the basis of fcc-type crystal arrangement. The strong diffraction peak at 38.21° is ascribed to the {111} facet of the fcc-metal gold structure. The other two peaks can be attributed to {200} and {220} facets at 44.19° and 64.45°, respectively. It is important to note that the ratio of intensity between {200} and {111} peaks is lower than the standard value (0.47 versus 0.53). Also, the ratio between {220} and {111} peaks is lower than the standard value (0.32 versus 0.33). These observations indicate that gold nanoplates (and not nanospheres, although both will exhibit circular plane) were formed in majority by the reduction of Au(III) by membrane-bound fraction of *E. coli* K12 and are dominated by {111} facets. Further, most of the {111} planes parallel to the surface of the supporting substrate were sampled.

### Catalytic activity of Au-MBF biocatalyst in 4-nitrophenol degradation

Aqueous 4-NP shows maximum UV–vis absorbance at 317 nm [26]. When NaBH4 (pH > 12) was added to reduce 4-NP, an intense yellow colour appeared due to formation of 4-nitrophenolate ion red-shifting the absorption peak to 400 nm [27]. The reaction does not proceed, and the peak remained for several days in the absence of Au catalyst [28,29]. Also, no peak change was observed in the control reaction consisting of MBF only without Au NPs.

**Figure 6:**
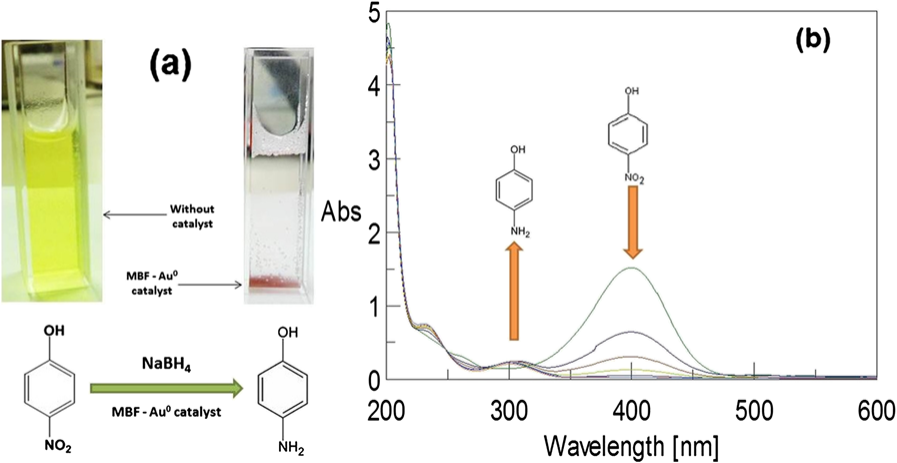
**Degradation of 4-nitrophenol and UV–vis absorption spectra.** (**a**) Schematic representation of degradation of 4-nitrophenol from pale yellow into colourless solution in the presence of MBF-Au^0^ heterogeneous catalyst; (**b**) UV–vis absorption spectra during the reduction of 4-nitrophenolate ion by Au NPs bound to MBF over a time period of 10 min.

Normally, -NO_2_-containing aromatic compounds are inert to the reduction via NaBH_4_. However, with the addition of MBF-Au NP biocatalyst, the colour faded to a colourless solution (as shown in Figure 6a) and the peak at 400 nm decreases with the appearance of the peak at 290 nm corresponding to the formation of 4-AP [30]. Au NPs present in the biocomposite helped in the transfer of electron from BH_4_ ^−^ ions to the nitro group of 4-NP and reducing it to 4-AP, which was qualitatively monitored by UV–vis spectroscopy as shown in Figure 6b. Since the concentration of bionanocomposite catalysing the reaction was very low, measurement of the absorption spectra of 4-NP and the reduction product 4-AP was not disturbed by the light scattering due to the catalyst carrier particles in the reaction mixture. As the concentration of NaBH_4_ used was much higher than that of 4-NP, it is assumed that the concentration of BH_4_ ^−^ remains constant during the reaction, and in this context, the order of reaction can be considered as a pseudo-first-order reaction [31]. We found good linear correlation of ln(*A*) and time, and the kinetic reaction rate constant under the given set of reaction conditions was estimated to be 1.24 × 10^−2^ min^−1^. However, it should be noted that the reduction rate of 4-NP can be influenced by the concentration of catalyst, size of catalyst, concentration of reactants and temperature [32]. Here, we observed that the biomass-supported catalyst proved to be a sturdy substitute for catalyst matrix as biogenic nanoparticles tend to adhere/adsorb to the biomaterial matrix because of certain active chemical groups, which in turn may impart additional stability to the biocatalyst framework. Further, the biomass alone in the absence of Au NPs was inert to the reaction. This ‘green catalyst’ will greatly reduce the cost incurred in bioremediation with an added advantage of being a totally eco-friendly synthesis process. Although there may be a few drawbacks like polydispersity of nanoparticles which may affect the quality of nanobiocatalyst, nonetheless considering the economic viability and facile green synthesis, this study helps in better understanding of bacteria-mediated nanoparticle synthesis and associated development of biocatalysts for the reduction of nitroaromatic pollutants.

## Conclusions

Extracellular membrane fraction of *E. coli* K12 was found to be responsible for the biogenic synthesis of gold nanoparticles at room temperature without pH adjustment. Gold nanoplates were obtained in majority in the size range of around 50 nm. We concluded that certain proteins embedded in the membrane fraction cause formation and stabilization of Au NPs. In the absence of these proteins (activity loss by β-met treatment), no nanoparticle formation was observed. Since biogenic nanoparticles are stabilized ‘naturally’ in the presence of active biomass, their efficacy in the preparation of heterogeneous catalyst was examined. We provided an innovative approach to utilize biogenic gold nanoparticles adsorbed over the cell membrane fraction to be used as a heterogeneous catalyst for catalysing complete degradation of 4-NP. A distinct advantage of this study lies in the fact that the facile green synthesis process can be seamlessly aligned with the preparation of nanobiocatalyst which may find numerous applications in catalysis, bioremediation studies, etc. This research has the potential to promote membrane fractions (proteins) for continuous synthesis of different types of NPs (see Additional file 1) and subsequent development of associated bionanocomposite resulting in improved material synthesis and application by biogenic systems.

## Abbreviations

4-AP: 4-aminophenol;4-NP: 4-nitrophenol;β-met: β-mercaptoethanol;MBF: membrane-bound fraction;NP: nanoparticle

## Competing interests

The authors declare that they have no competing interests.

## Authors’ contributions

SKS designed the protocol, carried out the experimental analysis and drafted the manuscript. CO and RY provided necessary technical discussions along with manuscript development. AK supervised the research and provided necessary infrastructural support. All authors have read and approved the final manuscript.

## Supplementary Material

Additional file 1 **Supplementary information.** It contains information about SDS-PAGE and preparation of membrane-bound fraction (MBF) column reactor for continuous synthesis of Au NPs. Click here for file
