# Supplementary material for: Biogenic synthesis and characterization of gold nanoparticles by Escherichia coli K12 and its heterogeneous catalysis in degradation of 4-nitrophenol
Source: Nanoscale Res Lett. 2013 Feb 12;8(1):70. doi: 10.1186/1556-276X-8-70 (PMC3599219; doi:10.1186/1556-276X-8-70)
Supplement: Additional file 1 — Supplementary information. It contains information about SDS-PAGE and preparation of membrane-bound fraction (MBF) column reactor for continuous synthesis of Au NPs. [file 1556-276X-8-70-S1.pdf]

SUPPLEMANTARY INFORMATION

Biogenic synthesis and characterization of gold nanoparticles by *Escherichia coli* K12 and its heterogeneous catalysis in degradation of 4-nitrophenol

Sarvesh Kumar Srivastava[1], Ryosuke Yamada[2], Chiaki Ogino[1], Akihiko Kondo[1]*

[1]Department of Chemical Science and Engineering, Graduate School of Engineering, Kobe University, 1-1 Rokkodai-cho, Nada, Kobe, 657-8501, Japan.

[2]Organization of Advanced Science and Technology, Kobe University, 1-1 Rokkodai-cho, Nada, Kobe, 657-8501, Japan.

*E-mail: akondo@kobe-u.ac.jp

Tel/Fax: +81-(0)78-803-6196

As discussed in the manuscript, SDS-PAGE analysis showed strong evidence of membrane bound proteins responsible for Au NP production. After a series of experimentations, we excised the SDS-PAGE gel protein lanes and extracted all the proteins bands as per ‘Passive Elution of Proteins from Polyacrylamide Gel Pieces’ protocol. These extracted protein fractions were tested for the reduction of $HAuCL_4^-$ into Au^0.

Membrane bound fraction showed several protein fractions as shown in Fig S1b. This is compared to a protein ladder (Precision Plus™ Protein Standard 250 KDa) in figure S1a. Subsequently, each of the visible protein bands was excised as mentioned above. After a completing protein separation and purification steps, these membrane protein fractions were tested for their reduction potential by their introduction in $HAuCl_4$ solution (0.01 M). We observed that the protein fraction of size ~73KDa in particular, has a strong effect on reducing $HAuCL_4^-$ into Au^0 along with a smaller protein fraction of size around 25 KDa which may be responsible for nanoparticle stabilization as highlighted in figure S1d. This 25 KDa protein was also found to be ‘wrapped’ around the Au NPs as described in the manuscript. Further, as discussed in the manuscript, when the reaction mixture was treated with β-met most of the secondary amide peaks were disappeared (FT-IR) suggesting loss of catalytic activity by these proteins as no nanoparticle formation was observed.

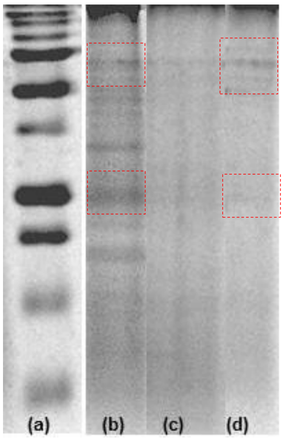

Figure S1. SDS-PAGE gel lanes showing (a) Precision plus™ protein ladder in comparison with (b) membrane bound fraction proteins, (c) membrane bound fraction treated with β-met and (d) protein fraction of size around 25KDa and 75KDa which showed strong sing for Au NP synthesis and stabilization.

Since, microbial mediated nanoparticle synthesis has several advantages over chemical methods and needs to compete for a continuous bulk-scale production process. Therefore, we tried producing biogenic Au NPs by constructing a ‘continuous reactor’ prototype where reduction is achieved by feeding gold cation solution from one end and obtaining Au nanoparticles from the other end of the reactor column. Au NP synthesis column was prepared by extracting MBF as described previously and packing it with silica in a microcolumn (Fig. S2a) in ratio 1:10 (w/w). It was expected that membrane bound proteins will cause reduction of gold ions yielding nanoparticle solution from the other end of the column. In this experiment, 10 mL of 0.01M HAuCl4 solution was passed through a column consisting of membrane bound fraction (MBF) of *E. coli* K12 and silica packed tightly in a column. Likewise, control reaction consisted of silica

packed column only and gold cationic solution without any inoculum. It was observed that the reaction mixture showed visible color change after 30 minutes and the solution collected from column indicated presence of Au NP due to color change from pale yellow to light pink (Fig. S2b) due to SPR while no such result was observed in control reactions.

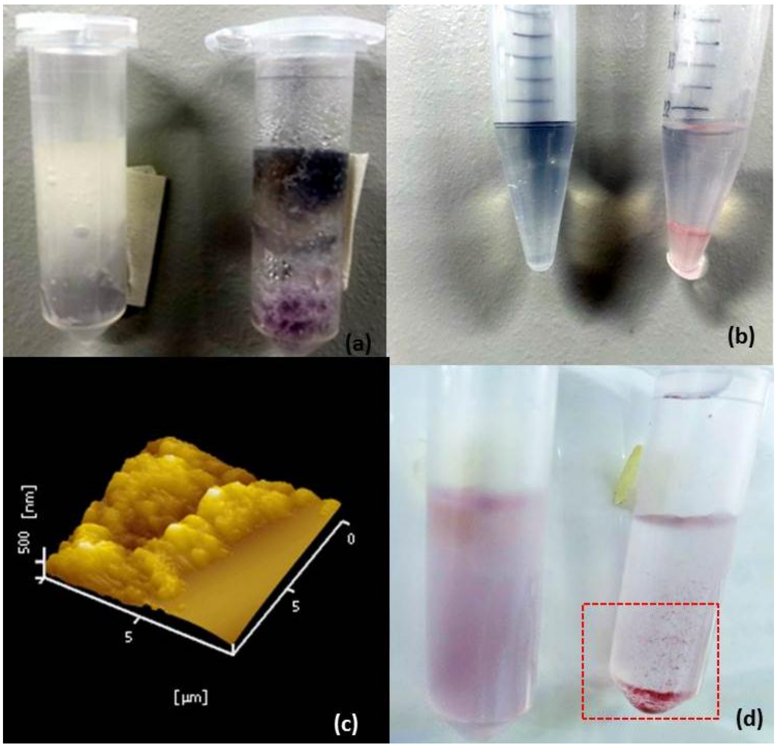

Fig. S2 Incorporation of MBF of *E. coli* K12 with silica in a micro-column: (L to R)

(a) silica column without MBF after passage of gold cation solution, silica column column with MBF after passage of gold cation solution; (b) visible color change in the cation solution passed through MBF bound column due to SPR by formation of Au NPs (c) AFM visualisation of Au NP produced by MBF-silica column d particle aggregation (indicated region) in MBF-silica column as compared to MBF reaction mixture.

As expected, resulting nanoparticles showed certain degree of aggregation (Fig. S2d) as observed visually since there was no incorporation of stabilizing agent to stabilize resulting nanoparticles. This was confirmed by zeta-potential analysis where mean Z-pot came out to be -0.47 mV suggesting particles are instable in solution. AFM analysis (Fig. S2c) showed greater aggregation of nanoparticles in the range of 250 - 500 nm by our crude 'biogenic-nanoparticle column reactor'. Particle size can be greatly controlled by incorporating capping agents like SDS and glycerol in collection beaker. We are currently examining column properties based on its regeneration potential, nanoparticle yield and means to obtain monodisperse NPs.

Finally, this technique can also be used to 'enrich' the biomass with desired nanoparticles in preparation of different types of heterogeneous catalytic bionanocomposites. This study will greatly benefit the ever-growing field of Biogenic synthesis and its subsequent development in biomimetic synthesis.
